# SLOB, a SLOWPOKE Channel Binding Protein, Regulates Insulin Pathway Signaling and Metabolism in *Drosophila*

**DOI:** 10.1371/journal.pone.0023343

**Published:** 2011-08-05

**Authors:** Amanda L. Sheldon, Jiaming Zhang, Hong Fei, Irwin B. Levitan

**Affiliations:** Department of Neuroscience, Thomas Jefferson University, Philadelphia, Pennsylvania, United States of America; New Mexico State University, United States of America

## Abstract

There is ample evidence that ion channel modulation by accessory proteins within a macromolecular complex can regulate channel activity and thereby impact neuronal excitability. However, the downstream consequences of ion channel modulation remain largely undetermined. The *Drosophila melanogaster* large conductance calcium-activated potassium channel SLOWPOKE (SLO) undergoes modulation via its binding partner SLO-binding protein (SLOB). Regulation of SLO by SLOB influences the voltage dependence of SLO activation and modulates synaptic transmission. SLO and SLOB are expressed especially prominently in median neurosecretory cells (mNSCs) in the *pars intercerebralis* (PI) region of the brain; these cells also express and secrete *Drosophila* insulin like peptides (dILPs). Previously, we found that flies lacking SLOB exhibit increased resistance to starvation, and we reasoned that SLOB may regulate aspects of insulin signaling and metabolism. Here we investigate the role of SLOB in metabolism and find that *slob* null flies exhibit changes in energy storage and insulin pathway signaling. In addition, *slob* null flies have decreased levels of *dilp3* and increased levels of *takeout*, a gene known to be involved in feeding and metabolism. Targeted expression of SLOB to mNSCs rescues these alterations in gene expression, as well as the metabolic phenotypes. Analysis of fly lines mutant for both *slob* and *slo* indicate that the effect of SLOB on metabolism and gene expression is via SLO. We propose that modulation of SLO by SLOB regulates neurotransmission in mNSCs, influencing downstream insulin pathway signaling and metabolism.

## Introduction

Large conductance, calcium- and voltage-sensitive potassium channels (BK channels) play a critical role in the regulation of neuronal excitability and neurotransmitter release. The *Drosophila melanogaster* BK channel is encoded by the *slowpoke* (*slo*) gene [1], [2]. SLO binds to and is regulated by several different protein kinases [3], [4], as well as by a novel protein named SLOB (for Slo-binding) that we isolated by a yeast two-hybrid screen using the extended carboxyl-terminal tail domain of SLO as bait [5], [6]. SLOB is expressed especially prominently in median neurosecretory cells (mNSCs) in the *pars intercerebralis* (PI) region of the fly brain [7]. SLOB expression level in these neurons modulates whole-cell potassium current and properties of single SLO channels [8]. Because these neurons also express and secrete *Drosophila* insulin-like peptides (dILPs) [7], [9], we hypothesized that SLOB might influence behavior related to feeding or metabolism. Indeed, *slob* mutant fly lines with dramatically decreased SLOB expression show prolonged survival under conditions of complete food-deprivation [8]; such a change in survival under starvation conditions is thought to reflect differences in feeding behavior and/or metabolism during the period prior to food-deprivation [10]. In addition, *slob* null flies exhibit altered locomotor activity during starvation (Reddy and Levitan, unpublished results). Wild-type flies typically exhibit an extended period of activity under starvation conditions; such hyperactivity is thought to reflect an adaptive foraging strategy in response to diminished food availability [11]. *Slob* null flies lack this hyperactive response, suggesting a role for the SLO/SLOB complex in mNSCs in integrating food stimuli and coordinating a response to nutrient conditions.

Altered insulin/insulin-like growth factor signaling (IIS) has also been implicated in increased resistance to starvation [12], [13]. The IIS pathway is conserved throughout evolution and is a critical regulator of growth, development, and lifespan (reviewed in [14]). Seven insulin-like peptides are expressed in *Drosophila melanogaster*: dILP 1–7 [15]. Of these, *dilp2*, *dilp3*, and *dilp5* are expressed in mNSCs of adult flies [9], [12], which project to the corpora cardiaca (CC), a pair of neurohemal glands located on the walls of the aorta [16]. CC cells express adipokinetic hormone (AKH), which is similar to mammalian glucagon; the PI-CC system in fruit flies is functionally homologous to the hypothalamic-pituitary axis in mammals [17]. MNSCs also project to the dorsal blood vessel, allowing for direct release of dILPs into the circulating hemolymph [16]. Together, AKH and dILPs regulate the levels of circulating sugars [16], [18], [19]. Disruption of the insulin receptor (InR) or mNSC ablation causes developmental delay, growth retardation, elevated levels of triglycerides, and increased levels of circulating glucose and trehalose [15], [16], [20], [21].

Interestingly, *slob* and *slo* are both regulated in a circadian manner [7], [22]. Other circadian genes have also been implicated in metabolism. For example *takeout* (*to*) encodes a protein similar to juvenile hormone binding protein and also cycles with a daily rhythm [10], [23]. *To* is expressed in structures related to feeding, such as the cardia, crop, antennae, and head fat body [10], [23], [24]. Similar to the mammalian liver, the fat body is the storage site for lipids and glycogen in insects [25]. *To* mutant flies are hyperphagic and exhibit alterations in energy storage [24]. Furthermore, they are more sensitive to starvation [10].

Here we sought to determine how SLOB expression in mNSCs influences insulin pathway signaling and metabolism. We find that *slob* mutant flies exhibit alterations in downstream measures of insulin signaling, as well as differences in energy storage. In addition, we present evidence that lack of SLOB in mNSCs results in dramatic changes in gene expression of *to* and *dilp3*. Interestingly, the effect of SLOB on metabolism appears to depend on SLOB's effect on *to* expression level. Importantly, intact SLO function is necessary for changes manifested in the *slob* null phenotype, implying that the modulation of SLO by SLOB mediates the alterations in gene expression and metabolism.

## Results

### SLOB levels regulate expression of *to*

**Figure 1:**
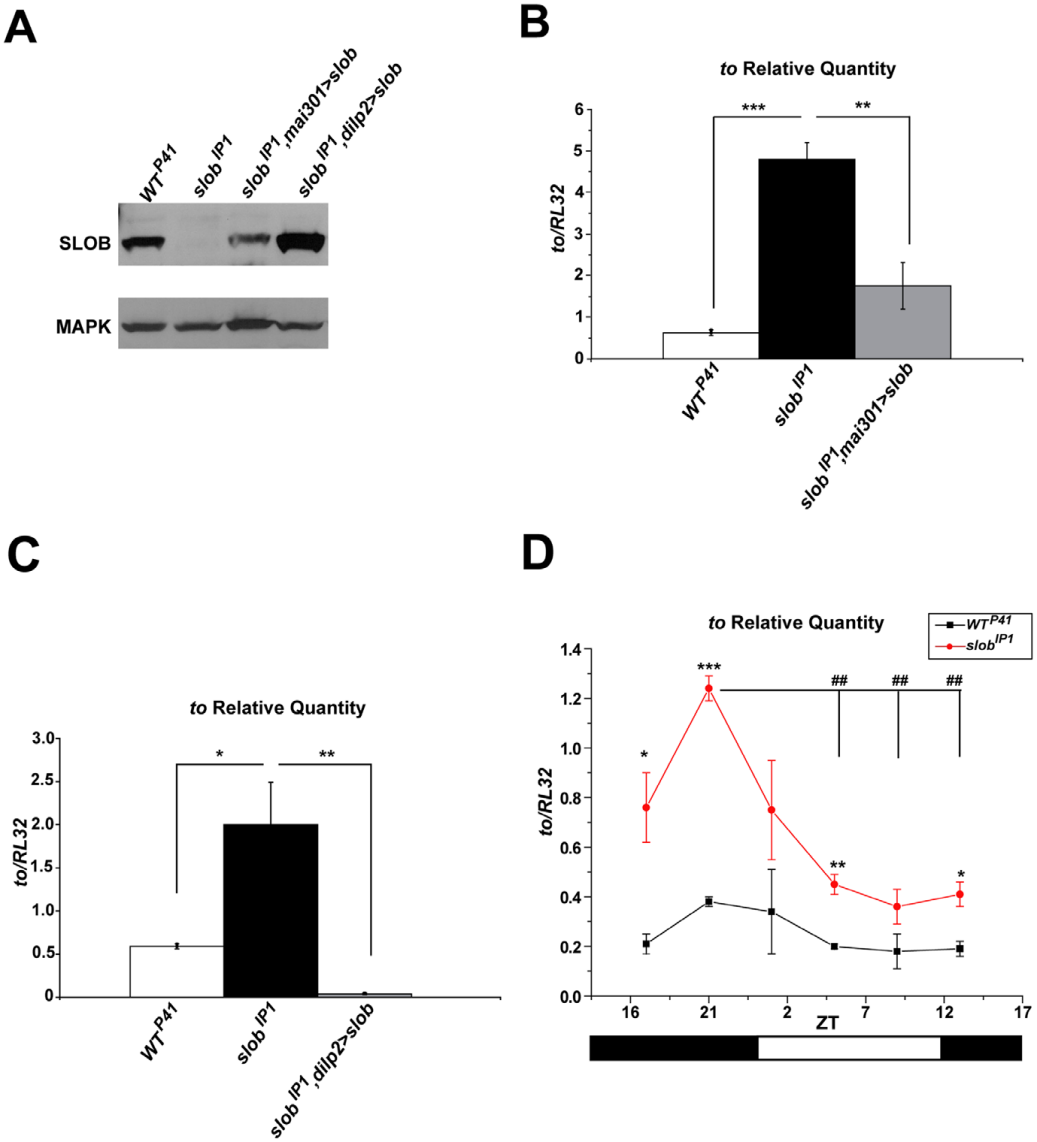
*to* mRNA levels are increased in *slob^IP1^* fly heads but still cycle. ***A,*** Western blot demonstrating rescue of SLOB expression in fly heads using two separate drivers for mNSCs in the *slob^IP1^* background. ***B*** **,** ***C*** **,** *to* mRNA levels in fly heads were measured by qPCR. *to* relative transcript levels are increased in *slob^IP1^* flies and rescued by targeted expression of *slob* in mNSCs. ***D*** **,** *to* transcript levels cycle in *WT^P41^* and *slob^IP1^* fly heads. Zeitgeber time (ZT) is plotted on the X axis; the white and black bars represent “lights on” and “lights off”, respectively. Each graph is a summary of a minimum of three independent experiments (mean ± SEM). For comparisons between fly lines, * indicates p<0.05, ** indicates p<0.01, *** indicates p<0.001; for comparisons between ZT points within one fly line, ## indicates p<0.01, One-way ANOVA with Bonferroni post-test.

**Figure 2:**
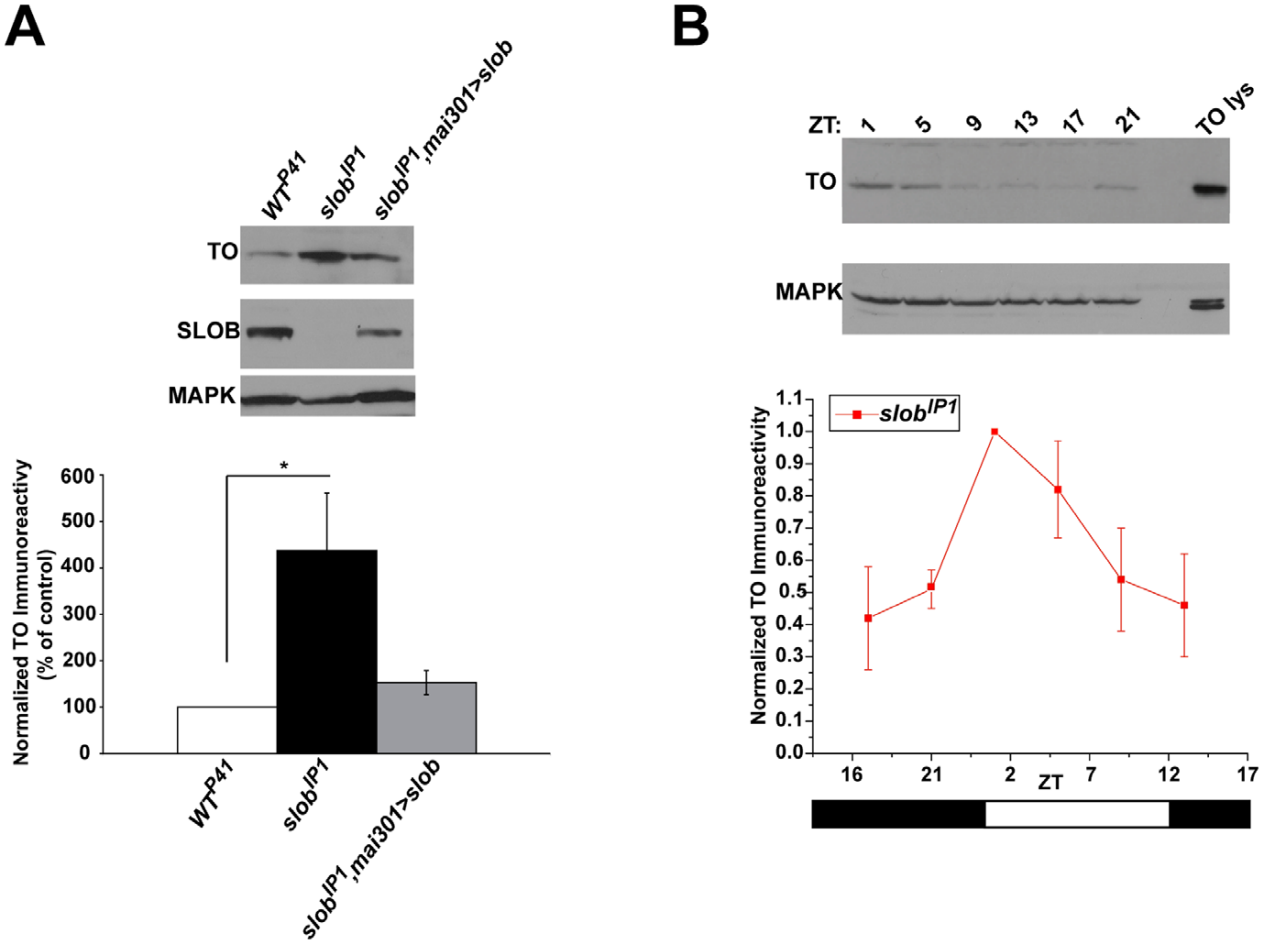
TO protein levels are increased in *slob^IP1^* fly heads but still cycle. TO protein levels in fly heads were measured by Western blot analysis and normalized to MAPK levels. ***A*** **,** Representative Western blot showing an increase in TO expression in *slob^IP1^* fly heads compared to *WT^P41^* fly heads. TO levels are rescued in *slob^IP1^,mai301>slob* fly heads. The graph is a summary of four independent experiments (mean ± SEM). * indicates p<0.05, One-way ANOVA with Bonferroni post-test. ***B*** **,** Representative Western blot showing that TO levels cycle in *slob^IP1^* fly heads. Zeitgeber time (ZT) is plotted on the X axis; the white and black bars represent “lights on” and “lights off”, respectively. The graph is a summary of three independent experiments (mean ± SEM).

The circadian protein TO is involved in regulation of feeding and energy storage; in addition, *to^1^* mutant flies exhibit decreased resistance to starvation [10], [23], [24]. Since *slob* null flies also exhibit a starvation phenotype, surviving significantly longer than wild-type control flies during starvation stress [8], we sought to determine whether *to* expression is altered in *slob* null flies. To this end, *to* transcript levels were measured in control (*WT^P41^*) and *slob* null (*slob^IP1^*) fly heads, as well as in rescue lines expressing SLOB specifically in mNSCs. Two rescue lines in the *slob^IP1^* background were examined. The *mai301*-GAL4 driver targets expression to mNSCs, as well as some additional neurons [26], whereas the *dilp2*-GAL4 driver line is specific for the *dilp*-expressing mNSCs [16], [27]. SLOB expression is restored with either driver (Fig. 1A). Interestingly, *to* transcript levels are significantly upregulated in *slob^IP1^* fly heads, and this effect is rescued by expression of SLOB in mNSCs, regardless of the driver (Fig. 1B, C). The *slob^IP1^, dilp2>slob* line expresses the least amount of *to* and this correlates with the highest levels of SLOB expression (Fig. 1A, C), suggesting a role for SLOB in regulating *to* levels. SLOB expression is also regulated in a circadian manner [7], [22]; therefore, we next investigated whether cycling of *to* transcripts is disrupted in flies lacking SLOB. As previously reported, *to* levels peak around ZT 17–21 (Fig. 1D) [10]. *To* transcript levels are elevated at all time points in *slob^IP1^* fly heads; however, cycling of *to* remains intact in *slob^IP1^* fly heads under LD conditions. To determine if upregulation of *to* persists at the protein level, fly head lysates were run on Western blots and probed for TO. *Slob^IP1^* flies express significantly more TO protein than *WT^P41^* flies, and this effect is also rescued by expression of SLOB in mNSCs (Fig. 2A). In addition, TO protein levels still cycle in *slob^IP1^* fly heads (Fig. 2B).

**Figure 3:**
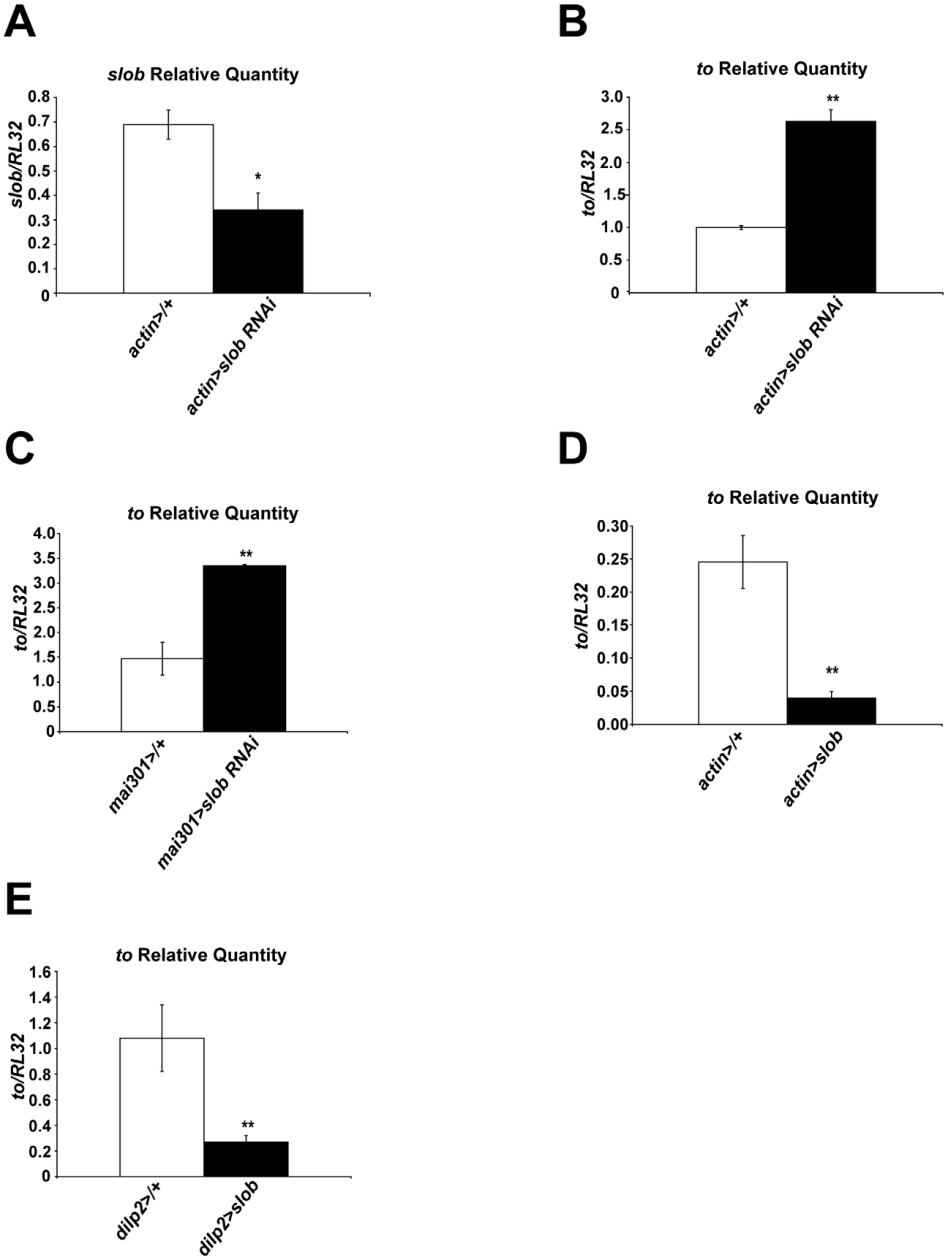
*to* mRNA levels are dependent on expression of SLOB. *Slob* and *to* levels in fly heads were measured by qPCR. ***A*** **,** Relative *slob* transcript levels are decreased in heads of flies in which SLOB expression was knocked down ubiquitously using RNAi. ***B***, Relative *to* transcript levels are increased in heads of flies in which SLOB expression was knocked down ubiquitously using RNAi. ***C*** **,** Relative *to* transcript levels are increased in heads of flies in which SLOB expression was knocked down in mNSCs using RNAi. ***D*** **,** Relative *to* transcript levels are decreased in heads of flies in which there is ubiquitous overexpression of SLOB. ***E*** **,** Relative *to* transcript levels are decreased in heads of flies in which SLOB was overexpressed exclusively in mNSCs. Each graph is a summary of a minimum of three independent experiments (mean ± SEM). * indicates p<0.05, ** indicates p<0.01, Student's t-test.

**Figure 4:**
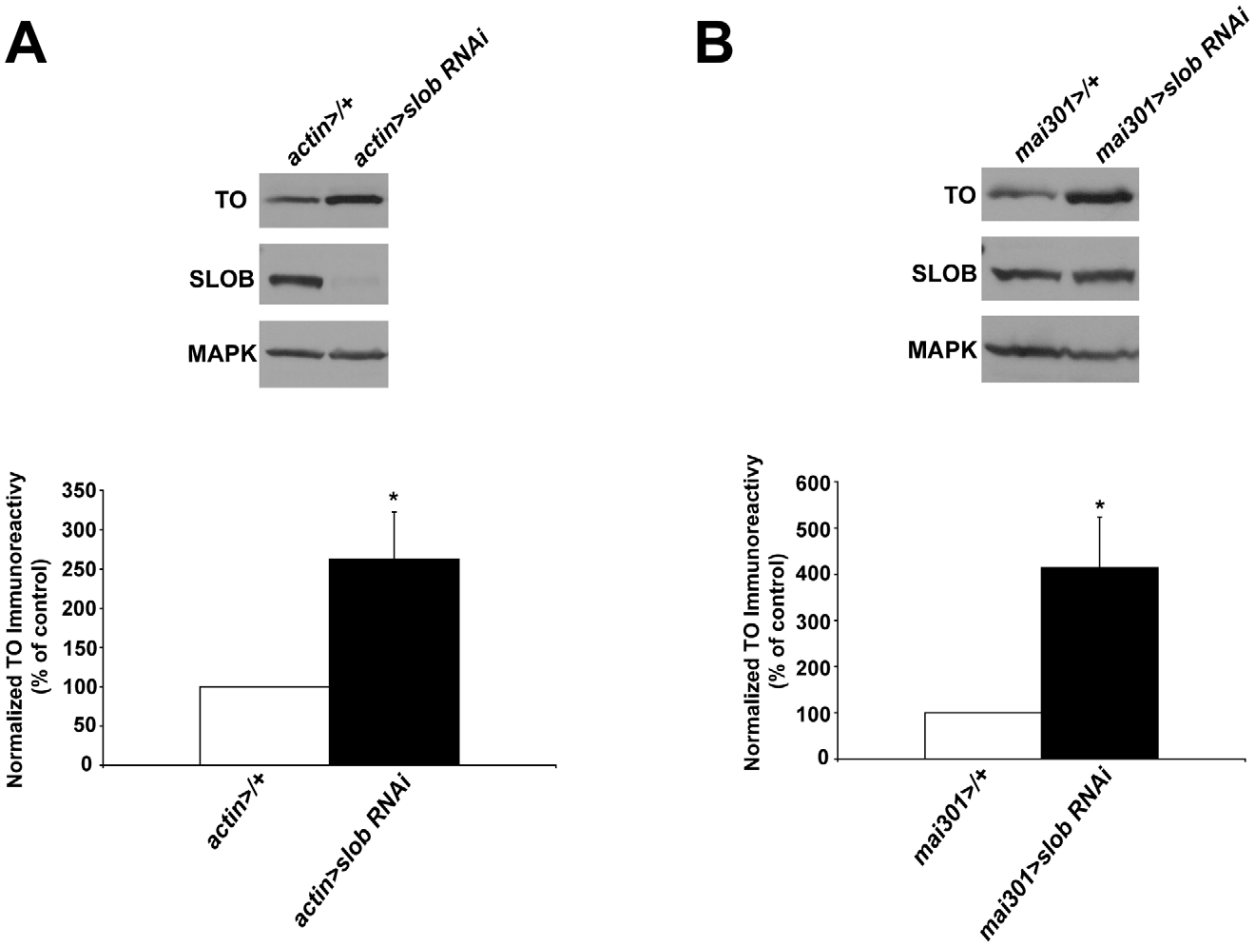
TO protein levels are dependent on expression of SLOB. TO protein levels in fly heads were measured by Western blot analysis and normalized to MAPK levels. ***A*** **,** Representative Western blot showing increased TO in heads of flies in which SLOB expression was knocked down ubiquitously using RNAi. The graph is a summary of five independent experiments (mean ± SEM). ***B*** **,** Representative Western blot showing increased TO in heads of flies in which SLOB expression was knocked down in mNSCs using RNAi. The graph is a summary of six independent experiments (mean ± SEM). * indicates p<0.05, One-way ANOVA with Bonferroni post-test.

In order to confirm that SLOB levels in mNSCs regulate the expression level of *to*, we used a different method to decrease SLOB expression, namely *slob*-RNAi (Fig. 3A). Ubiquitous knockdown of SLOB results in significantly increased levels of *to* in fly heads (Fig. 3B). Furthermore, knockdown of SLOB in mNSCs is also sufficient to increase expression of *to* (Fig. 3C). Conversely, ubiquitous overexpression of SLOB results in significantly decreased transcript levels of *to* (Fig. 3D), and overexpression of SLOB in mNSCs only is sufficient to decrease *to* transcript levels (Fig. 3E). Likewise, ubiquitous knockdown of SLOB results in increased levels of TO protein (Fig. 4A), and targeted knockdown of SLOB in mNSCs also causes elevated levels of TO (Fig. 4B). Unsurprisingly, total SLOB protein levels are not decreased in *mai301>slob RNAi* fly heads because SLOB is expressed in other cell types such as photoreceptors, which are not targeted by the *mai301* driver [7]. Therefore it appears that SLOB levels in mNSCs influence expression levels of TO, although the mechanism underlying this effect is unclear.

### SLOB levels regulate expression of *dilp3*

**Figure 5:**
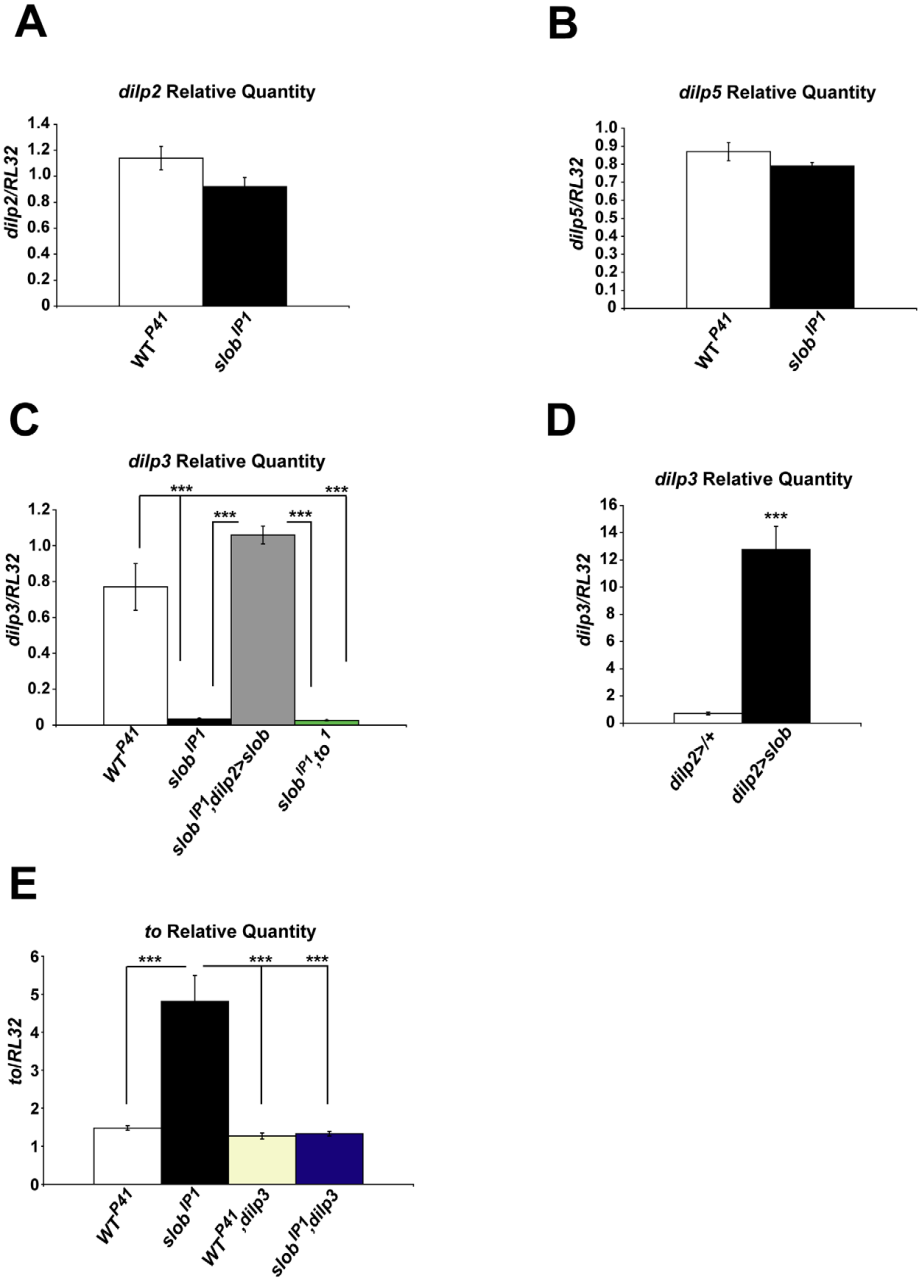
Effect of SLOB on expression of *dilps*. Relative *dilp2*, -*3*, and -*5* transcript levels in fly heads were measured by qPCR. ***A, B*** **,** Relative *dilp2* or *dilp5* transcript levels are unchanged in *slob^IP1^* fly heads. ***C*** **,** Relative *dilp3* transcript levels are reduced in *slob^IP1^* fly heads and rescued by expression of SLOB in mNSCs, but not by mutation of *to*. ***D,*** Relative *dilp3* transcript levels are increased in heads of flies overexpressing SLOB in mNSCs. ***E,*** The increase in *to* levels is abolished in *slob^IP1^,dilp3* fly heads. Each graph is a summary of a minimum of three independent experiments (mean ± SEM). *** indicates p<0.001, One-way ANOVA with Bonferroni post-test (*C, E*) or Student's t-test (*D*).

Since SLOB is expressed especially prominently in mNSCs [7], we hypothesized that SLOB may influence IIS by modifying dILP expression or release. DILP2 is the most abundant dILP expressed by mNSCs and has the greatest effect on carbohydrate metabolism [12]. Although *dilp2* transcript levels are slightly decreased in *slob^IP1^* fly heads, we found no significant difference in *dilp2* levels between *WT^P41^* and *slob^IP1^* fly heads (Fig. 5A, Table 1). Likewise, levels of *dilp5* transcript are not significantly different between *WT^P41^* and *slob^IP1^* fly heads (Fig. 5B). However, there is a dramatic reduction in *dilp3* levels in *slob^IP1^* fly heads compared to *WT^P41^* fly heads (Fig. 5C, Table 1), and this effect is rescued by expression of SLOB in mNSCs only (Fig. 5C). Conversely, overexpression of SLOB in mNSCs results in a striking upregulation of *dilp3* (Fig. 5D), suggesting that expression levels of SLOB in mNSCs regulate *dilp3* levels. The slight decreases in *dilp2* and *dilp5* transcript levels are consistent with a role for *dilp3* as a positive regulator of *dilp2* and *dilp5* expression [28]. Since TO is significantly increased in *slob^IP1^* fly heads, and TO is expressed in the head fat body, which has been shown to signal to mNSCs [29], [30], we sought to determine whether TO can influence *dilp3* expression. To this end, we measured *dilp3* transcripts in the double mutant line *slob^IP1^,to^1^*. Interestingly, expression of *dilp3* remains low in *slob^IP1^,to^1^* fly heads; hence TO is not required for downregulation of *dilp3* in *slob^IP1^* fly heads (Fig. 5C). Alternatively, *dilp3* levels may influence *to* expression; in order to determine if the effect of SLOB on *dilp3* expression is upstream of the effect on *to* expression, we generated fly lines lacking *dilp3* in the *WT^P41^* or *slob^IP1^* background. We find that *to* is expressed at equivalent levels in *WT^P41^*, *WT^P41^,dilp3*, and *slob^IP1^,dilp3* fly heads, indicating that *dilp3* is not required for basal expression of *to* (Fig. 5E). However, the upregulation of *to* exhibited by *slob^IP1^* fly heads is abolished in *slob^IP1^,dilp3* fly heads; therefore, even though *dilp3* is greatly reduced in *slob^IP1^* flies, it appears that the minimal residual amount of *dilp3* is required for the control of *to* expression by SLOB levels.

**Table 1:** Measures of gene expression and metabolism in *slob^IP1^* flies, expressed as a percentage of *WT^P41^* control levels.

	*slob^IP1^* (% of *WT^P41^*)
*to* Relative Quantity	475%
TO protein expression	383%
*dilp2* Relative Quantity	81%
*dilp3* Relative Quantity	4%
*dilp5* Relative Quantity	91%
Circulating trehalose and glucose	43%
Whole body trehalose and glucose	43%
P-AKT expression	190%
Triglycerides	137%

### *Slob* null flies exhibit alterations in energy metabolism and insulin pathway signaling

**Figure 6:**
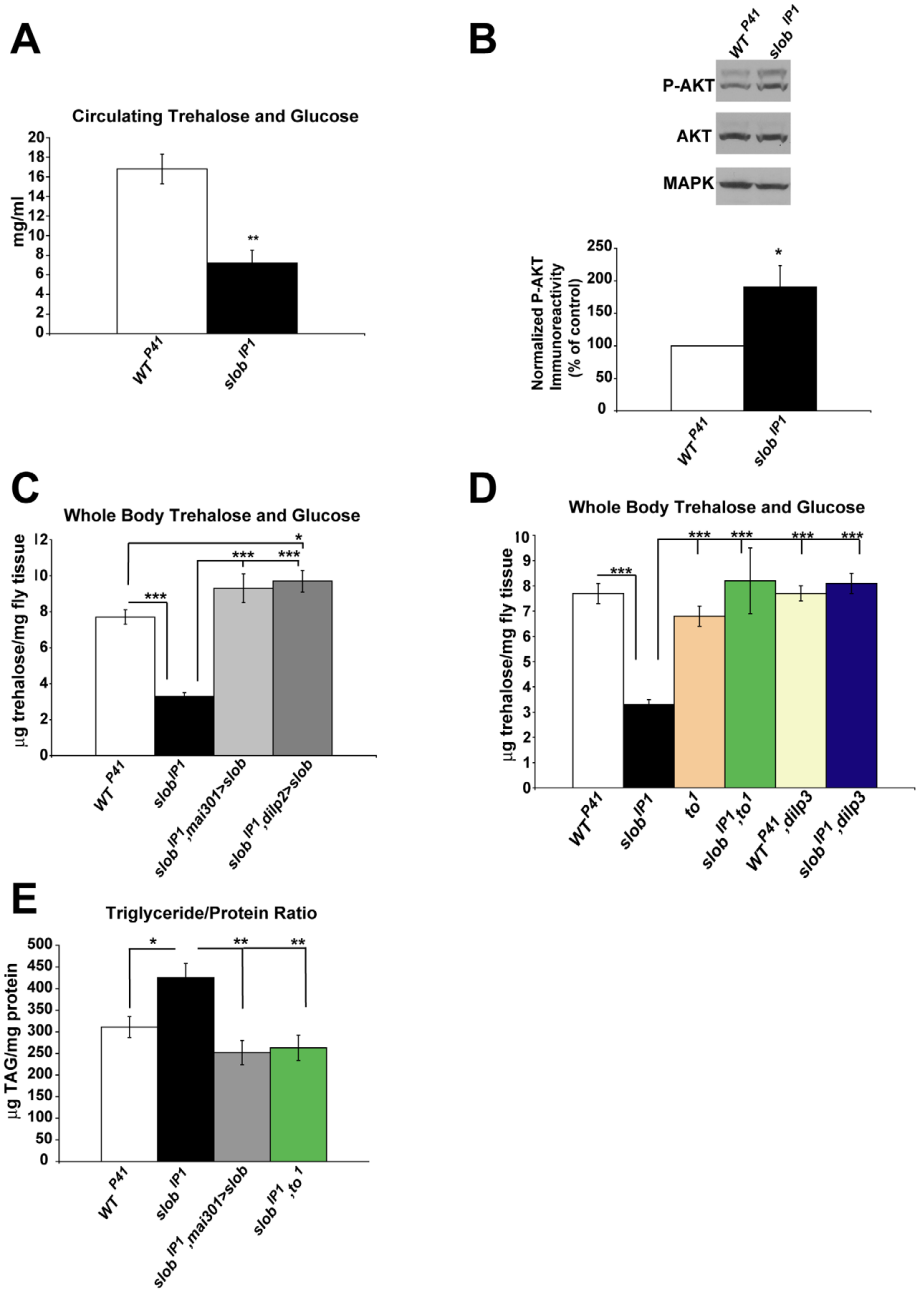
*slob^IP1^* flies exhibit alterations in energy metabolism and insulin pathway signaling. ***A*** **,** Hemolymph was extracted from flies after fasting, and circulating trehalose and glucose were measured. *Slob^IP1^* flies display significantly decreased levels of circulating trehalose plus glucose in hemolymph. Results are a summary of a minimum of five independent experiments (mean ± SEM). ** indicates p<0.01, Student's t-test. ***B*** **,** P-AKT levels were measured by Western blot and normalized to MAPK. Representative Western blot showing an increase in P-AKT in *slob^IP1^* fly heads compared to *WT^P41^* fly heads. The graph is a summary of eight independent experiments (mean ± SEM). * indicates p<0.05, One sample t-test. ***C*** **,** Whole body trehalose plus glucose levels were measured in flies after fasting. Stored trehalose plus glucose is decreased in *slob^IP1^* flies and rescued by expression of SLOB in mNSCs. ***D*** **,** Whole body trehalose plus glucose levels are restored in fly lines with decreased levels of *to* compared to *slob^IP1^* flies, including *dilp3* fly lines. Each stored trehalose graph is a summary of a minimum of six samples (mean ± SEM). * indicates p<0.05, *** indicates p<0.001, One-way ANOVA with Bonferroni post-test. ***E*** **,** Whole body lipid levels were measured and normalized to protein levels. *Slob^IP1^* flies display increased storage of triglycerides compared to *WT^P41^* flies, and this effect is rescued by expression of SLOB in mNSCs or by mutation of *to* in the *slob^IP1^* background. Results are presented as a summary of a minimum of nine samples (mean ± SEM). * indicates p<0.05, ** indicates p<0.01, One-way ANOVA with Bonferroni post-test.

SLOB binds to and modulates SLO activity in mNSCs [8]; in addition, we recently demonstrated that flies lacking SLOB exhibit enhanced neurotransmission at the neuromuscular junction, and this is due to modulation of SLO by SLOB in the presynaptic nerve terminal [31]. We therefore hypothesized that *slob^IP1^* flies might exhibit altered excitability in mNSC terminals, resulting in differences in dILP release and IIS pathway signaling. Altered IIS is associated with changes in energy storage; mNSC ablation or mutation of genes encoding key components of the IIS pathway results in fasting hyperglycemia and altered triglyceride storage [12], [15], [16], [20], [21], [32]. In addition, *to* mutant flies exhibit altered energy metabolism [24], therefore we investigated levels of sugars and triglycerides in *slob^IP1^* flies. Circulating carbohydrates in the fly consist of trehalose, the main homeostatic sugar, and glucose from the diet [33]. *Slob^IP1^* flies exhibit significantly decreased levels of circulating trehalose and glucose (Fig. 6A and Table 1), indicative of elevated IIS in *slob* null flies. To assess activation of the IIS pathway more directly, phosphorylated AKT (P-AKT) was measured in *WT^P41^* and *slob^IP1^* fly heads. InR activation results in downstream phosphorylation of AKT at Ser 505 in *Drosophila*, which is homologous to phosphorylation of AKT at Ser 473 in mammals [34]. P-AKT is significantly upregulated in *slob^IP1^* flies (Fig. 6B), indicating increased activation of IIS, while total AKT levels remain the same; both PAKT and AKT levels were normalized to levels of the loading control MAPK in these experiments. Although activation of MAPK is altered by IIS, total MAPK levels remain the same [35]–[37]; therefore normalizing P-AKT to MAPK is appropriate. We also normalized P-AKT levels to ACTIN levels, and this analysis yielded results similar to those obtained by normalizing P-AKT to MAPK (data not shown). Measuring circulating sugars in adult flies is difficult due to the small volume of hemolymph present. Therefore we sought to determine if measures of stored sugars in *WT^P41^* and *slob^IP1^* flies reflect those of circulating sugars. Indeed, whole body trehalose and glucose levels are also decreased in *slob^IP1^* flies compared to *WT^P41^* flies (Fig. 6C, Table 1), again suggesting enhanced IIS in *slob^IP1^* flies. Levels of stored trehalose and glucose are rescued in transgenic flies expressing SLOB under the control of mNSC-targeted drivers, *mai301*-GAL4 or *dilp2*-GAL4, indicating that expression of SLOB in mNSCs is sufficient to restore whole body trehalose levels. Stored trehalose is also restored in *slob^IP1^,to^1^* flies, as well as in single *to* mutants (*to^1^*) (Fig. 6D). Therefore it appears that one mechanism underlying the effect of SLOB on whole body sugars is through SLOB's regulation of *to* expression. In support, both fly lines lacking *dilp3*: *WT^P41^,dilp3* and *slob^IP1^,dilp3*, express equivalent levels of *to* and exhibit whole body sugar levels similar to those of *WT^P41^* flies (Fig. 5E, 6D). Whole body trehalose levels are unchanged in single *dilp3* mutants [28]; similarly, we find that *WT^P41^,dilp3* flies have levels of whole body trehalose and glucose comparable to those of *WT^P41^* flies. However, the decrease in stored sugars exhibited by *slob^IP1^* is abolished in *slob^IP1^,dilp3* flies; this result is consistent with lack of upregulation of *to* in *slob^IP1^,dilp3* flies.

Triglyceride levels are altered in *to^1^* flies, as well as in IIS pathway mutant flies [24], [38]. Also, flies which exhibit increased resistance to starvation often display increased storage of triglycerides [39]–[41]. Likewise, *slob^IP1^* flies exhibit increased storage of triglycerides compared to *WT^P41^* flies, and this effect is rescued by expression of SLOB in mNSCs (Fig. 6D). Interestingly, triglyceride levels are also rescued in *slob^IP1^,to^1^* flies, indicating that the effect on lipid metabolism requires TO.

### SLO is required for the effects on gene expression and metabolism in *slob* null flies

**Figure 7:**
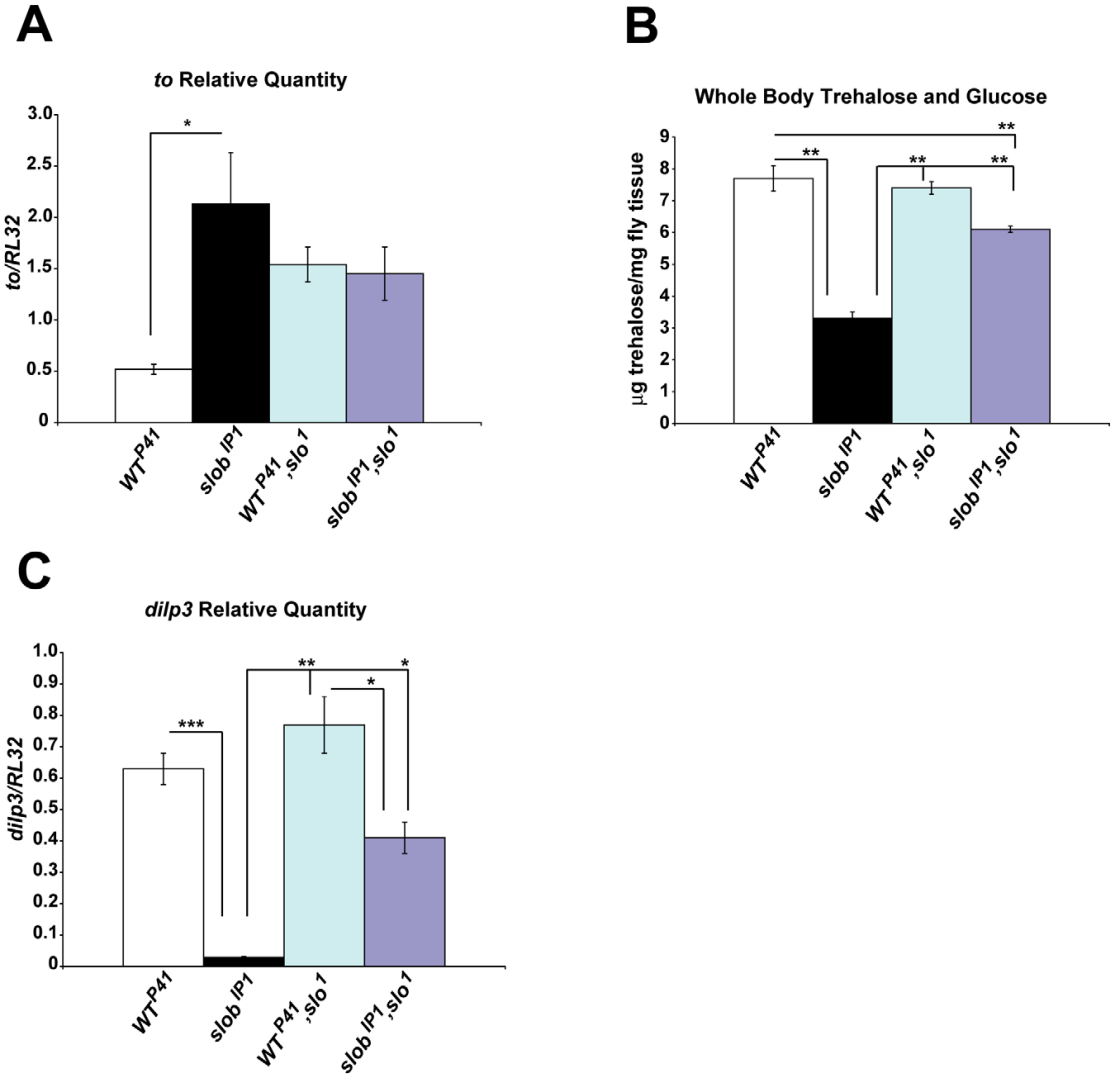
The effects of SLOB on gene expression and metabolism require SLO. ***A,*** Relative *to* transcript levels in fly heads were measured by qPCR. *WT^P41^,slo^1^* and *slob^IP1^,slo^1^* express equivalent amount of *to* in fly heads. ***B,*** Whole body trehalose plus glucose levels were measured in flies after fasting. Stored trehalose plus glucose levels are significantly decreased in *slob^IP1^* flies compared to *WT^P41^,slo^1^* or *slob^IP1^,slo^1^* flies. ***C,*** The reduction in *dilp3* levels is attenuated in *slob^IP1^,slo^1^* fly heads. Results are a summary of a minimum of three (*A, C*) or 13 samples (*B*) (mean ± SEM). * indicates p<0.05, ** indicates p<0.01, *** indicates p<0.001, One-way ANOVA with Bonferroni post-test.

We have established that modulation of SLO activity by SLOB influences neuronal excitability and neurotransmitter release [8], [31]. In order to determine if the effects of SLOB on gene expression and metabolism are dependent on modulation of SLO by SLOB, we crossed the mutant *slo^1^* line [1] into the *WT^P41^* and *slob^IP1^* fly lines to generate *WT^P41^,slo^1^* and *slob^IP1^,slo^1^* lines. *WT^P41^,slo^1^* and *slob^IP1^,slo^1^* flies express approximately equivalent amounts of *to* in fly heads at levels intermediate to those in *WT^P41^* and *slob^IP1^* flies (Fig. 7A). Compared to *slob^IP1^*, there is a trend towards decreased levels of *to* in these *slo^1^* mutant lines, suggesting that the effect of SLOB on *to* expression may be due at least in part to modulation of SLO. Interestingly, intact SLO function is not a requirement for *to* expression, as demonstrated by expression of *to* in *WT^P41^,slo^1^* flies. However, SLO function is necessary for the upregulation of *to* due to the lack of SLOB, as there is no increase in *to* in *slob^IP1^,slo^1^* fly heads compared to *WT^P41^,slo^1^* fly heads. Similarly, *WT^P41^,slo^1^* and *slob^IP1^,slo^1^* flies exhibit increased amounts of whole body sugars compared to *slob^IP1^*, supporting a role for modulation of SLO in SLOB's effect on metabolism (Fig. 7B).

We also measured *dilp3* levels in *WT^P41^,slo^1^* fly heads, and found *dilp3* levels unchanged in *WT^P41^,slo^1^* fly heads compared to *WT^P41^* fly heads. Since the SLOB present in *WT^P41^* heads acts to inhibit SLO function [8], [42], and SLO activity is not intact in *WT^P41^,slo^1^* flies due to the *slo^1^* mutation, these results suggest that decreased SLO activity results in greater levels of *dilp3*. Conversely, SLO activity is elevated in *slob^IP1^* flies [8], and *dilp3* expression is much reduced (Fig. 7C). Although lack of SLOB still results in decreased expression of *dilp3* in the *slo^1^* mutant line (compare *WT^P41^,slo^1^* and *slob^IP1^,slo^1^*), the effect is significantly attenuated compared to *dilp3* downregulation in *slob^IP1^* heads. Therefore, the effect of SLOB on *dilp3* expression functions at least in part through modulation of SLO.

## Discussion

Previously, we found that *slob* null flies live significantly longer than control flies under conditions of complete food deprivation [8]. Increased resistance to starvation is often accompanied by changes in energy storage or alterations in genes involved in metabolism. We find that levels of the metabolic gene *to* are upregulated almost five-fold in *slob* null flies, and this effect is rescued by expression of SLOB targeted specifically to mNSCs. Using RNAi to decrease SLOB levels results in a similar increase, even when SLOB knockdown is targeted to mNSCs. Furthermore, overexpression of SLOB either ubiquitously or specifically in mNSCs downregulates *to* levels. We confirmed that TO protein levels are similarly upregulated in *slob* null flies. The mechanism underlying regulation of *to* in response to SLOB levels in mNSCs is unclear. Since mNSCs project to the CC and the dorsal blood vessel, it is likely that there are several steps between SLOB regulation of mNSCs and its downstream effects on *to* expression in areas of the head such as the fat body. Like *slob*, levels of *to* are regulated by the circadian clock [10], [23]. Although *to* levels are elevated at all time points in *slob* null flies, *to* still cycles in the absence of SLOB, suggesting that factors other than SLOB influence circadian regulation of *to*. PAR domain protein 1 (PDP1ε) is a circadian transcription factor reported to indirectly regulate *to* expression [43]; however, *pdp1ε* transcript levels are not significantly different between control and *slob* null flies (data not shown). TO protein levels also cycle in *slob* null flies. Expression levels were measured under LD conditions however, and TO protein expression is directly regulated by light [43]; therefore it is not surprising that TO still cycles in the absence of SLOB. It would be interesting to examine whether TO protein still cycles in constant darkness in *slob* null flies.

It has been proposed that TO may link circadian and feeding behaviors. Of note, *to* mutant flies die faster during starvation [10]; in combination with our results showing that SLOB expression level regulates *to* expression, we conclude that upregulation of TO in *slob* null flies mediates starvation resistance. In addition, *slob* null flies exhibit increased storage of triglycerides, which may enable them to withstand longer durations of starvation. *To* mutant flies are hyperphagic and also exhibit increased energy storage; however they cannot harness this energy during periods of starvation, resulting in early death [10], [24]. Decreasing TO levels in the *slob* null background, either through SLOB rescue in mNSCs or through mutation of *to*, restores triglyceride levels to those of control flies, suggesting that metabolic changes due to the lack of SLOB are mediated by TO. Likewise, fly lines which exhibit low levels of *to* relative to *slob* null flies also exhibit increased levels of trehalose compared to *slob* null flies, suggesting that TO regulates sugar levels as well. The mechanism underlying TO-mediated changes in metabolism is still not entirely clear; TO is secreted into the hemolymph and shares sequence similarity with juvenile hormone binding protein (JHBP) [10]. However it is unknown if TO is also a carrier for juvenile hormone (JH); in addition the receptor for JH remains poorly understood.

**Figure 8:**
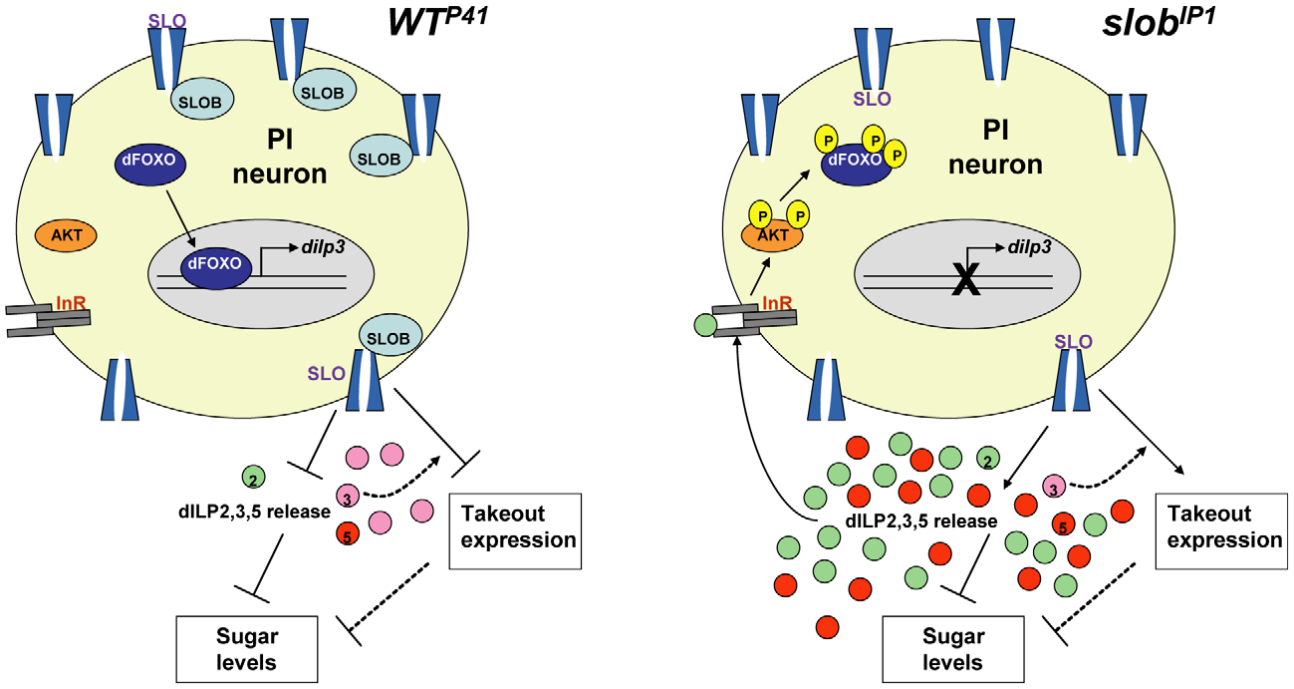
Proposed function of SLOB in mNSCs. SLOB, through inhibitory modulation of SLO, influences dILP release from mNSCs and gene expression of *to* and *dilp3*. *Slob* null flies have elevated SLO activity, resulting in opposite effects on IIS and gene expression. Arrows denote positive regulation, while blunted lines denote negative regulation. Dotted lines denote relationships that are still unclear. See text for further details.

Since SLOB is co-expressed with dILPs in mNSCs [7], we reasoned that SLOB may influence the IIS pathway, perhaps by regulating expression or release of dILPs. DILP2 has the most profound effect on IIS pathway activation and metabolism; however there is no significant change in *dilp2* expression in *slob* null flies. Surprisingly, we discovered a striking decrease in *dilp3* levels in *slob* null flies. This effect is due to SLOB in mNSCs, since it is rescued by specific expression of SLOB in mNSCs. Conversely, overexpression of SLOB in mNSCs results in dramatically upregulated levels of *dilp3*, suggesting that SLOB in mNSCs positively regulates *dilp3* expression. How might changes in SLOB expression regulate *dilp3* transcript levels? *Dilp3* transcription undergoes autocrine regulation, whereby dILP signaling through InRs expressed by mNSCs results in downstream sequestration of the transcription factor dFOXO to negatively regulate transcription of *dilp3*, but not *dilp2* or *dilp5* [44]. It follows that *dilp3* expression will be downregulated by elevated IIS resulting from increased release of dILPs from mNSCs (Fig. 8). Several lines of evidence suggest that IIS activity is indeed enhanced in *slob* null flies. First, circulating sugars in *slob* null flies are less than half those of control flies. In addition, whole body trehalose and glucose levels are similarly decreased in *slob* null flies. The effect of SLOB on carbohydrate metabolism is mediated by SLOB in mNSCs, since two independent drivers for expression of SLOB in mNSCs restore sugar levels to those of control flies. Finally, phosphorylation of AKT is increased in *slob* null flies, indicating activation of InRs by circulating dILPs.

There is a high degree of compensation among dILPs, and mutation of *dilp3* alone has no effect on varied measures of the IIS pathway [28]. As levels of *dilp2* and *dilp5* are not significantly altered, it is unlikely that downregulation of *dilp3* alone in *slob* null flies can account for changes in circulating and stored sugars; rather our data suggest that release of dILPs from mNSCs is increased in *slob* null flies, resulting in enhanced IIS pathway activation. This conclusion is consistent with reports that *dilp3* transcription is under control of an autocrine feedback loop [28], [44]. Although disrupted IIS is often associated with increased storage of triglycerides [12], [13], [16], [21], studies demonstrating elevated IIS resulting in increased levels of triglycerides have also been reported [32], [45], [46]. Therefore, increased triglyceride storage in *slob* null flies is not at odds with elevated IIS in these flies.

Expression levels of *dilp2*, *dilp3*, and *dilp5* are differentially regulated by nutrition, stress, and genetic manipulations [9], [12], [47]–[49]. For instance, starvation suppresses expression of *dilp3* and *dilp5*, but not *dilp2* [9], while altering the nutrient composition of food by lowering the concentration of yeast reduces expression of *dilp5* alone [47]. On the other hand, *dilp2* expression is downregulated by the stress-activated Jun-N-terminal kinase pathway [48]. Although we have evidence for increased IIS pathway signaling in *slob* null flies, it is unknown which DILP is mediating downstream effects on metabolism. *Dilp2* and *dilp5* are both abundant in *slob* null flies, and it would be interesting to examine individual production or release of *dilp2* vs. *dilp5*.

It is clear that SLOB expression level greatly influences expression of *to* and *dilp3*, as well as metabolic measures. The fat body signals to mNSCs via an unknown secreted factor to influence dILP release [29]; in addition, the fat body is a target of circulating dILPS released by mNSCs, so we wondered if alterations in *slob* mutant flies were dependent on either *to* or *dilp3*. Whole body trehalose and glucose levels are restored to wild-type levels by mutation of *to* in the *slob* null background. Although *to* is necessary for the metabolic alterations manifested in *slob* null flies, *to* is not required for the downregulation of *dilp3*. Similarly, flies lacking *dilp3* alone exhibit levels of *to* equivalent to those of wild-type flies. On the other hand, *dilp3* is required for the upregulation of *to* expression displayed by flies lacking SLOB. Therefore, although *to* and *dilp3* are independently regulated by SLOB, *dilp3* modulates the effect of SLOB on *to* expression and resultant downstream metabolic measures (Fig. 8). In addition to *dilps* and *to*, other genes involved in regulation of metabolism have been identified, including *adipokinetic hormone*, *target of brain insulin*, *slimfast*, , *hugin*, *klumpfuss*, and *pumpless* [11], [50]–[54]. We hypothesize that expression of some of these genes will also be altered in *slob* null flies; therefore, expression of key metabolic genes in *slob* null flies will be investigated in future experiments.

In mammals, glucose-induced insulin secretion is primarily regulated by ATP sensitive K^+^ channels (K_ATP_) in pancreatic islet beta cells (reviewed in [55]). Increased ATP due to glucose metabolism inhibits K_ATP_ channels, resulting in beta cell depolarization and insulin secretion. Adult fruit flies express the sulfonylurea receptor (Sur) subunit of the K_ATP_ channel in mNSCs [56], [57]; in addition, mNSCs are sensitive to glucose and glibenclamide, a K_ATP_ blocker, demonstrating that adult mNSCs display electrophysiological properties similar to beta cells. Interestingly, a role for BK channels in mammalian insulin secretion has recently come back into favor. BK channel knock-out mice exhibit broadened single action potentials in beta cells and reduced glucose-stimulated insulin release [58]. Similarly, we have shown that inhibition of SLO by SLOB [6] increases action potential duration in mNSCs [8]. In addition, we recently demonstrated that synaptic transmission is reduced when SLOB is present, and SLO activity is thereby inhibited; conversely, neurotransmitter release is enhanced in *slob* null flies, wherein SLO activity is elevated [31]. Decreased circulating and stored sugars in *slob* null flies are consistent with enhanced release of dILPs from mNSCs (Fig. 8). It is appealing to speculate that mammalian BK channels may undergo similar modulation by associated proteins to influence insulin release. Additionally, we present evidence that the effects of SLOB on metabolism, upregulation of *to*, and downregulation of *dilp3* are dependent on modulation of SLO. Taken together, these results suggest that modulation of SLO by SLOB mediates the effects of SLOB on metabolism and gene expression.

## Materials and Methods

### *Drosophila* stocks and maintenance

Fruit flies were cultured at 25°C on standard *Drosophila* medium and maintained on a 12∶12 hr light∶dark cycle. For all experiments, female flies were separated from males after eclosion and then collected for experimentation 5 days post-eclosion at Zeitgeber time 5 (ZT 5). Generation of wild-type control (*WT^P41^*), *slob* null (*slob^IP1^*), *slob* rescue flies expressing *slob* in mNSCs (*slob^IP1^,mai301>slob*), UAS-*slob*, and UAS-*slob*-RNAi flies in the *yw* background was described in detail previously [8], [31]. In brief, *slob^IP1^* flies were obtained from the imprecise excision of a P-element insertion in the *slob* gene; they express no SLOB. The *WT^P41^* fly line was obtained from precise excision of the P-element and serves as a control for the *slob^IP1^* line. *Slob^IP1^* flies do not exhibit any gross growth or developmental defects. Multiple isoforms of *slob* exist [59], but *slob57* is the most prominent isoform. In these studies *slob57* was used for expression or RNAi knockdown and is referred to simply as *slob* in these lines. *Actin*-GAL4, *mai301*-GAL4, and *ry506,to^1^* (*to^1^*) lines were provided by Dr. Amita Sehgal (University of Pennsylvania), and *dilp2*-GAL4 by Dr. Eric Rulifson (University of California, San Francisco). *To^1^* flies have a deletion in the 3′ region of *to* genomic DNA, resulting in very low levels of basal expression and rendering mutants incapable of regulating *to* expression in response to starvation [10]. *St^1^slo^1^* (*slo^1^*) and *dilp3* [28] lines were from the Bloomington Stock Center. No molecular characterization of *slo^1^* is available, but it has been used extensively as a *slo* mutant line and exhibits electrophysiological and circadian phenotypes [1], [22], [60]–[62]. *Dilp3* is undetectable by qPCR in *dilp3* mutant flies (data not shown). *Dilp2*-GAL4 and *dilp3* lines were outcrossed into the *yw* background seven times and a series of crosses were then conducted to generate the following lines: *WT^P41^,dilp3*; *slob^IP1^, dilp3*; and *slob^IP1^,dilp2>slob*, a second rescue line expressing *slob* specifically in insulin producing neurons in the PI region (mNSCs). Additional crosses were conducted to create the following lines: *WT^P41^,slo^1^*; *slob^IP1^,slo^1^*; and *slob^IP1^,to^1^* lines. UAS-*slob* and UAS-*slob*-RNAi lines were maintained as homozygous lines and crossed to GAL4 driver lines prior to experimentation.

### Quantitative RT-PCR (qPCR)

Total RNA was extracted from a minimum of 30 fly heads using the UltraSpec RNA isolation system following the manufacturer's recommendations (Biotecx Laboratories). 2 µg of RNA was reverse transcribed using the High Capacity RNA-cDNA kit (Applied Biosystems). QPCR was performed on an Applied Biosystems 7000 detection system using Power SYBR green master mix and 1 ng template cDNA. Primers were designed using the Primer Express software (Applied Biosystems). Primer sequences are available upon request. Results were calculated for a minimum of three independent RNA extractions using the standard curve method and normalized to the ribosomal gene, *RL32*.

### Western blots

At least 30 fly heads were homogenized in lysis buffer containing 1% CHAPS, 20 mM Tris-HCl pH 7.5, 10 mM EDTA, 12 mM NaCl, 50 mM KCl, protease inhibitor cocktail (Sigma-Aldrich) and phosphatase inhibitor cocktail (Sigma-Aldrich) at 4°C. Flies were starved with 1% agar for 30 min prior to protein extraction for analysis of phosphorylated AKT (P-AKT), in order to establish a baseline regardless of food ingested immediately before protein extraction. Equivalent amounts of protein were separated on 4–12% Tris-Bis gradient gels and transferred to nitrocellulose blots. Blots were blocked with 5% nonfat milk in TBST (0.1% Tween in Tris-buffered saline) and probed with primary antibodies overnight. The following primary antibodies were used: rabbit polyclonal anti-SLOB [8], rat polyclonal anti-TO (kind gift of Dr. Michael Rosbash, Brandeis University [10]), rabbit polyclonal anti-*Drosophila* P-AKT (Cell Signaling Technology), rabbit polyclonal anti-pan AKT (Cell Signaling Technology), rabbit polyclonal anti-MAPK (Sigma-Aldrich), and rabbit polyclonal anti-β-actin (Cell Signaling). Following washes with TBST, blots were incubated with horseradish peroxidase-conjugated donkey anti-rabbit or anti-rat secondary antibody, washed again with TBST, and visualized using the Enhanced Chemiluminescence Detection System (GE Healthcare). The optical densities for proteins of interest were quantitated using NIH Image and normalized to the loading control MAPK. Furthermore, in 6 of 8 experiments in the PAKT data set, proteins were also normalized to β-actin.

### Trehalose and glucose measurements

To measure circulating trehalose and glucose, hemolymph was extracted by decapitation and centrifugation from adult female flies after a 5 hr starvation with 1% agar as previously described [12], [44]. 0.3 µL of hemolymph was added to 75 µL hexokinase reagent, pH 6.8 (ThermoElectron) in 96-well plates and incubated with 0.1 µL porcine trehalase (Sigma-Aldrich) at 37°C overnight to convert trehalose to glucose. Trehalose standards were similarly incubated with trehalase. Samples were measured in duplicate at 340 nm and compared to a standard curve. Whole body trehalose plus glucose was measured in adult female flies after an 18 hr starvation with 1% agar as previously described [63], [64]. Briefly, 10 flies per sample were weighed, crushed in 250 µL 0.24 M sodium carbonate, and incubated at 95°C for 2 hr to denature proteins. Samples were then mixed with 150 µL 1 M acetic acid and 600 µL 0.25 M sodium acetate, pH 5.2 and spun down at 12,500 rpm to pellet debris. 100 µL aliquot samples were incubated with 1 µL trehalase each overnight at 37°C. Trehalose standards of known concentrations underwent identical treatment. The following day, 10 µL of each sample was incubated with 90 uL hexokinase reagent (ThermoElectron) in triplicate in a 96-well plate at 37°C, measured at 340 nm, and compared to the standard curve. Sugars were normalized to the total mg of fly tissue in each sample.

### Lipid measurement

Triacylglycerides (TAG) were measured in adult female flies as described previously with slight modifications [45]. Briefly, 4 female flies were homogenized in 250 µL lysis buffer (140 mM NaCl, 50 mM Tris-HCl, pH 7.4, and 0.1% Triton-X) containing protease inhibitor cocktail (Sigma-Aldrich), sonicated, and then centrifuged at 12,500 rpm, 4°C. Protein and TAG were measured in supernatants using the BCA Protein Assay (Pierce) and Triglyceride Liquicolor (Stanbio) kits respectively, per manufacturer instructions. TAG was normalized to the amount of protein in each sample.

## References

[1] Atkinson NS, Robertson GA, Ganetzky B (1991). A component of calcium-activated potassium channels encoded by the Drosophila slo locus.. Science.

[2] Elkins T, Ganetzky B, Wu CF (1986). A Drosophila mutation that eliminates a calcium-dependent potassium current.. Proc Natl Acad Sci U S A.

[3] Wang J, Zhou Y, Wen H, Levitan IB (1999). Simultaneous binding of two protein kinases to a calcium-dependent potassium channel.. J Neurosci.

[4] Zhou Y, Wang J, Wen H, Kucherovsky O, Levitan IB (2002). Modulation of Drosophila slowpoke calcium-dependent potassium channel activity by bound protein kinase a catalytic subunit.. J Neurosci.

[5] Schopperle WM, Holmqvist MH, Zhou Y, Wang J, Wang Z (1998). Slob, a novel protein that interacts with the Slowpoke calcium-dependent potassium channel.. Neuron.

[6] Zeng H, Weiger TM, Fei H, Jaramillo AM, Levitan IB (2005). The amino terminus of Slob, Slowpoke channel binding protein, critically influences its modulation of the channel.. J Gen Physiol.

[7] Jaramillo AM, Zheng X, Zhou Y, Amado DA, Sheldon A (2004). Pattern of distribution and cycling of SLOB, Slowpoke channel binding protein, in Drosophila.. BMC Neurosci.

[8] Shahidullah M, Reddy S, Fei H, Levitan IB (2009). In vivo role of a potassium channel-binding protein in regulating neuronal excitability and behavior.. J Neurosci.

[9] Ikeya T, Galic M, Belawat P, Nairz K, Hafen E (2002). Nutrient-dependent expression of insulin-like peptides from neuroendocrine cells in the CNS contributes to growth regulation in Drosophila.. Curr Biol.

[10] Sarov-Blat L, So WV, Liu L, Rosbash M (2000). The Drosophila takeout gene is a novel molecular link between circadian rhythms and feeding behavior.. Cell.

[11] Lee G, Park JH (2004). Hemolymph sugar homeostasis and starvation-induced hyperactivity affected by genetic manipulations of the adipokinetic hormone-encoding gene in Drosophila melanogaster.. Genetics.

[12] Broughton SJ, Piper MD, Ikeya T, Bass TM, Jacobson J (2005). Longer lifespan, altered metabolism, and stress resistance in Drosophila from ablation of cells making insulin-like ligands.. Proc Natl Acad Sci U S A.

[13] Clancy DJ, Gems D, Harshman LG, Oldham S, Stocker H (2001). Extension of life-span by loss of CHICO, a Drosophila insulin receptor substrate protein.. Science.

[14] Goberdhan DC, Wilson C (2003). The functions of insulin signaling: size isn't everything, even in Drosophila.. Differentiation.

[15] Brogiolo W, Stocker H, Ikeya T, Rintelen F, Fernandez R (2001). An evolutionarily conserved function of the Drosophila insulin receptor and insulin-like peptides in growth control.. Curr Biol.

[16] Rulifson EJ, Kim SK, Nusse R (2002). Ablation of insulin-producing neurons in flies: growth and diabetic phenotypes.. Science.

[17] Veelaert D, Schoofs L, De LA (1998). Peptidergic control of the corpus cardiacum-corpora allata complex of locusts.. Int Rev Cytol.

[18] Isabel G, Martin JR, Chidami S, Veenstra JA, Rosay P (2005). AKH-producing neuroendocrine cell ablation decreases trehalose and induces behavioral changes in Drosophila.. Am J Physiol Regul Integr Comp Physiol.

[19] Kim SK, Rulifson EJ (2004). Conserved mechanisms of glucose sensing and regulation by Drosophila corpora cardiaca cells.. Nature.

[20] Chen C, Jack J, Garofalo RS (1996). The Drosophila insulin receptor is required for normal growth.. Endocrinology.

[21] Tatar M, Kopelman A, Epstein D, Tu MP, Yin CM (2001). A mutant Drosophila insulin receptor homolog that extends life-span and impairs neuroendocrine function.. Science.

[22] Ceriani MF, Hogenesch JB, Yanovsky M, Panda S, Straume M (2002). Genome-wide expression analysis in Drosophila reveals genes controlling circadian behavior.. J Neurosci.

[23] So WV, Sarov-Blat L, Kotarski CK, McDonald MJ, Allada R (2000). takeout, a novel Drosophila gene under circadian clock transcriptional regulation.. Mol Cell Biol.

[24] Meunier N, Belgacem YH, Martin JR (2007). Regulation of feeding behaviour and locomotor activity by takeout in Drosophila.. J Exp Biol.

[25] Van der Horst DJ (2003). Insect adipokinetic hormones: release and integration of flight energy metabolism.. Comp Biochem Physiol B Biochem Mol Biol.

[26] Siegmund T, Korge G (2001). Innervation of the ring gland of Drosophila melanogaster.. J Comp Neurol.

[27] Crocker A, Shahidullah M, Levitan IB, Sehgal A (2010). Identification of a neural circuit that underlies the effects of octopamine on sleep:wake behavior.. Neuron.

[28] Gronke S, Clarke DF, Broughton S, Andrews TD, Partridge L (2010). Molecular evolution and functional characterization of Drosophila insulin-like peptides.. PLoS Genet.

[29] Geminard C, Rulifson EJ, Leopold P (2009). Remote control of insulin secretion by fat cells in Drosophila.. Cell Metab.

[30] Hwangbo DS, Gershman B, Tu MP, Palmer M, Tatar M (2004). Drosophila dFOXO controls lifespan and regulates insulin signalling in brain and fat body.. Nature.

[31] Ma H, Zhang J, Levitan IB (2011). Slob, a Slowpoke channel-binding protein, modulates synaptic transmission.. J Gen Physiol.

[32] Zhang H, Liu J, Li CR, Momen B, Kohanski RA (2009). Deletion of Drosophila insulin-like peptides causes growth defects and metabolic abnormalities.. Proc Natl Acad Sci U S A.

[33] Klowden MJ (2002). Physiologicla Systems in Insects.

[34] Burgering BM, Coffer PJ (1995). Protein kinase B (c-Akt) in phosphatidylinositol-3-OH kinase signal transduction.. Nature.

[35] Kim SE, Cho JY, Kim KS, Lee SJ, Lee KH (2004). Drosophila PI3 kinase and Akt involved in insulin-stimulated proliferation and ERK pathway activation in Schneider cells.. Cell Signal.

[36] Kwon HB, Kim SH, Kim SE, Jang IH, Ahn Y (2002). Drosophila extracellular signal-regulated kinase involves the insulin-mediated proliferation of Schneider cells.. J Biol Chem.

[37] Lee KS, Kwon OY, Lee JH, Kwon K, Min KJ (2008). Drosophila short neuropeptide F signalling regulates growth by ERK-mediated insulin signalling.. Nat Cell Biol.

[38] Teleman AA (2010). Molecular mechanisms of metabolic regulation by insulin in Drosophila.. Biochem J.

[39] Sieber MH, Thummel CS (2009). The DHR96 nuclear receptor controls triacylglycerol homeostasis in Drosophila.. Cell Metab.

[40] Gronke S, Beller M, Fellert S, Ramakrishnan H, Jackle H (2003). Control of fat storage by a Drosophila PAT domain protein.. Curr Biol.

[41] Gronke S, Muller G, Hirsch J, Fellert S, Andreou A (2007). Dual lipolytic control of body fat storage and mobilization in Drosophila.. PLoS Biol.

[42] Zhou Y, Schopperle WM, Murrey H, Jaramillo A, Dagan D (1999). A dynamically regulated 14-3-3, Slob, and Slowpoke potassium channel complex in Drosophila presynaptic nerve terminals.. Neuron.

[43] Benito J, Hoxha V, Lama C, Lazareva AA, Ferveur JF (2010). The circadian output gene takeout is regulated by Pdp1epsilon.. Proc Natl Acad Sci U S A.

[44] Broughton S, Alic N, Slack C, Bass T, Ikeya T (2008). Reduction of DILP2 in Drosophila triages a metabolic phenotype from lifespan revealing redundancy and compensation among DILPs.. PLoS One.

[45] DiAngelo JR, Bland ML, Bambina S, Cherry S, Birnbaum MJ (2009). The immune response attenuates growth and nutrient storage in Drosophila by reducing insulin signaling.. Proc Natl Acad Sci U S A.

[46] DiAngelo JR, Birnbaum MJ (2009). Regulation of fat cell mass by insulin in Drosophila melanogaster.. Mol Cell Biol.

[47] Broughton SJ, Slack C, Alic N, Metaxakis A, Bass TM (2010). DILP-producing median neurosecretory cells in the Drosophila brain mediate the response of lifespan to nutrition.. Aging Cell.

[48] Wang MC, Bohmann D, Jasper H (2005). JNK extends life span and limits growth by antagonizing cellular and organism-wide responses to insulin signaling.. Cell.

[49] Bauer JH, Chang C, Morris SN, Hozier S, Andersen S (2007). Expression of dominant-negative Dmp53 in the adult fly brain inhibits insulin signaling.. Proc Natl Acad Sci U S A.

[50] Zinke I, Kirchner C, Chao LC, Tetzlaff MT, Pankratz MJ (1999). Suppression of food intake and growth by amino acids in Drosophila: the role of pumpless, a fat body expressed gene with homology to vertebrate glycine cleavage system.. Development.

[51] Buch S, Melcher C, Bauer M, Katzenberger J, Pankratz MJ (2008). Opposing effects of dietary protein and sugar regulate a transcriptional target of Drosophila insulin-like peptide signaling.. Cell Metab.

[52] Melcher C, Pankratz MJ (2005). Candidate gustatory interneurons modulating feeding behavior in the Drosophila brain.. PLoS Biol.

[53] Melcher C, Bader R, Pankratz MJ (2007). Amino acids, taste circuits, and feeding behavior in Drosophila: towards understanding the psychology of feeding in flies and man.. J Endocrinol.

[54] Colombani J, Raisin S, Pantalacci S, Radimerski T, Montagne J (2003). A nutrient sensor mechanism controls Drosophila growth.. Cell.

[55] Proks P, Lippiat JD (2006). Membrane ion channels and diabetes.. Curr Pharm Des.

[56] Fridell YW, Hoh M, Kreneisz O, Hosier S, Chang C (2009). Increased uncoupling protein (UCP) activity in Drosophila insulin-producing neurons attenuates insulin signaling and extends lifespan.. Aging (Albany NY).

[57] Kreneisz O, Chen X, Fridell YW, Mulkey DK (2010). Glucose increases activity and Ca(2+) in insulin-producing cells of adult Drosophila.. Neuroreport.

[58] Dufer M, Neye Y, Horth K, Krippeit-Drews P, Hennige A (2011). BK channels affect glucose homeostasis and cell viability of murine pancreatic beta cells.. Diabetologia.

[59] Jaramillo AM, Zeng H, Fei H, Zhou Y, Levitan IB (2006). Expression and function of variants of slob, slowpoke channel binding protein, in Drosophila 1.. J Neurophysiol.

[60] Atkinson NS, Brenner R, Chang W, Wilbur J, Larimer JL (2000). Molecular separation of two behavioral phenotypes by a mutation affecting the promoters of a Ca-activated K channel.. J Neurosci.

[61] Gho M, Ganetzky B (1992). Analysis of repolarization of presynaptic motor terminals in Drosophila larvae using potassium-channel-blocking drugs and mutations.. J Exp Biol.

[62] Engel JE, Wu CF (1998). Genetic dissection of functional contributions of specific potassium channel subunits in habituation of an escape circuit in Drosophila.. J Neurosci.

[63] Belgacem YH, Martin JR (2006). Disruption of insulin pathways alters trehalose level and abolishes sexual dimorphism in locomotor activity in Drosophila.. J Neurobiol.

[64] Chen Q, Ma E, Behar KL, Xu T, Haddad GG (2002). Role of trehalose phosphate synthase in anoxia tolerance and development in Drosophila melanogaster.. J Biol Chem.

